# The Antiangiogenic Properties of Adipose-Derived Mesenchymal Stem/Stromal Cells in Corneal Neovascularization in a Rabbit Model

**Published:** 2020-03-12

**Authors:** Demetrios Pirounides, Anastasia Komnenou, Nikolaos Papaioannou, Eleni Gounari, Ioanna Stylianaki, Alexandros Alexandridis, Angeliki Chranioti, Evangelia Kofidou, Georgios Koliakos, Vasileios Karampatakis

**Affiliations:** 1 Department of Ophthalmology, Aristotle University of Thessaloniki, AHEPA University Hospital, Thessaloniki, Greece.; 2 Faculty of Veterinary Medicine, School of Health Sciences, Aristotle University of Thessaloniki, Thessaloniki, Greece.; 3 Medical School, Laboratory of Biological Chemistry, Aristotle University of Thessaloniki, Thessaloniki, Greece.; 4 Department of Ophthalmology, General Hospital of Karditsa, Karditsa, Greece.; 5 Faculty of Health Sciences, School of Medicine, Laboratory of Experimental Ophthalmology, Aristotle University of Thessaloniki, Thessaloniki, Greece.

**Keywords:** Adipose-derived Mesenchymal Stromal/stem Cells, Corneal Neovascularization, Anti-angiogenetic Properties, Suture-induced Corneal Neovascularization, Senescent Stem Cells.

## Abstract

The purpose was to study the anti-angiogenic effect of adipose-derived mesenchymal stem/stromal cells (ADMSCs) on experimentally induced corneal injuries. Corneal neovascularization (NV) was induced by incising and subsequently suturing the corneal surface in 32 New Zealand rabbits. Following suturing, the rabbits were randomly allocated into 2 groups, and received either phosphate-buffered saline (PBS) (control) or ADMSCs, both administered via three different routes. Digital images of the cornea were obtained two weeks post-incision to measure the area of neovascularized cornea. Tumor necrosis factor (TNF) was immunohistochemically assessed in the both groups. The corneal tissue was evaluated for vascular endothelial growth factor (VEGF). The extent of corneal NV in all eyes was assessed photographically by an independent observer. Fourteen days after the incisions, the degree of corneal NV was substantially decreased in the ADMSC-treated group (1.87 ± 0.9 mm^2^, 1.4 % ± 0.67 % of corneal surface) compared to the control and PBS-treated group (4.66 ± 1.74 mm^2^, 3.51 % ± 1.31 %, p < 0.001). ADMSCs significantly decreased injury-induced corneal NV in New Zealand rabbits two weeks post-treatment. This strategy has potential for use in the control of corneal NV *in vivo*.

## INTRODUCTION 

The cornea is the most important refractive structure of the eye due to its unique arrangement of histological components and hemispherical shape [1]. As an avascular tissue, the cornea is an immunologically privileged structure [2, 3], and owes its clarity to its unique arrangement of stromal collagen fibrils and lack of vascularization, at least centrally. Corneal transparency can be maintained through a dynamic balance between pro- and antiangiogenic mechanisms [4, 5]. Corneal neovascularization (NV) constitutes a defense mechanism against harmful agents [6] and diseases [7], and facilitates transfer of neutrophils, macrophages, dendritic cells, lymphocytes and cytokines closer to sites of injury [8]. During NV, endothelial cells proliferate, migrate and form new vessel branches [9]. Release of several angiogenetic mediators such as transforming growth factor (TGF) [10], galectin-3 [11], angiotensin and matrix metalloproteinases (MMPs) [12] contributes to multiple aspects of corneal NV [13]. Enhanced production of proangiogenic factors such as vascular endothelial growth factor (VEGF) [14, 15], angiopoietins [16] and tumor necrosis factor (TNF) favors angiogenesis [17]. After healing, the blood vessels leave empty structures known as ghost vessels [18].

Recently, it has been indicated that mesenchymal stem cells (MSCs) attenuate NV after insults such as burns and other injuries. These cells interact with host cells both directly or by paracrine signaling. Attenuated VEGF activity has been reported in MSC-treated rats [19], however, Watt et al. reported opposing results, suggesting that MSCs promote vasculogenesis/angiogenesis [20, 21]. To clarify this contradiction, we examined the antiangiogenic properties of MSCs in a rabbit model. 

Besides their reported antimicrobial effects, MSCs also exhibit adjuvant anti-inflammatory properties [22]. Neovascularization is inhibited through suppression of proangiogenic MMP-2 and induction of gene expression of the antiangiogenic factor thrombospondin-1 (TSP-1) [23], a VEGF inhibitor [19]. The corneal microenvironment promotes the antiangiogenic role of MSCs, which is important for optimal recovery without corneal scars and opacities. This is in contrast to other tissues where revascularization is also mediated by MSCs (i.e., the skin), where scarring is vital for the healing process [22]. 

In the present study, we focused on the antiangiogenic effects of adipose-derived MSCs (ADMSCs) in modifying the consequences of mechanical corneal injuries in a rabbit model.

## MATERIAL AND METHODS

**Culture of rabbit ADMSCs**

This interventional animal study was started in July 2015 and concluded in September 2018. ADMSCs were secluded as previously described [24]. Briefly, the animals were intramuscularly anesthetized by 30–50 mg/kg ketamine and 3–5 mg/kg xylazine (both from Lyon, France). Following surgical ablation from the inguinal region the fat pad was washed with phosphate-buffered saline and treated for 1 h at 37 °C with collagenase type 1 (Sigma–Aldrich, St Louis, The USA) under agitating. After introducing phosphate-buffered saline (PBS) to the tube which was left standing, the lower clear layer with ADMSCs was collected. 

The mesenchymal cells were cultured for no more than 5 passages (duplications) in a chamber with antibiotics.

**Characterization of the MSCs**

Identification of MSC’s specific markers was performed by flow cytometry. Briefly, cells were obtained with centrifugation and then stained against CD44 and CD73 (BD Pharmingen, The USA) [25]. A FACS Calibur device (Becton Dickinson, Franklin Lakes, The USA) was used. To test the differentiation capacity of MSCs, osteogenic or adipogenic medium (Thermo Fisher Scientific, MA, The USA) was added to the culture for approximately 30 days with a medium change every 3 days. Successful differentiation was assessed *via* alizarin red and oil red staining.

**Animal model**

Thirty-two New Zealand rabbits, each weighing 2500-3500 grams, were used in this experimental study. All the animals were kept in a specially designed, well-ventilated facility under stable temperature and humidity with a successive 12-hour light/dark cycle and fed ad libitum. All principles of the ARVO Statement were implemented [26]. All procedures and experimental designs of the study were reviewed and authorized by the appropriate state institutions (Ethics Committee and Committee of Veterinary Medicine of the Aristotle University of Thessaloniki). The study was conducted in the Surgery Unit of the School of Veterinary Medicine. Before the study, all the rabbits underwent a thorough ophthalmic examination to exclude those with ocular pathology. 

A previously described model of penetrating injury [27, 28] was modified to assess the effects of MSCs on corneal wounding and NV. All the animals were anesthetized by intramuscular injection of 75–100 μg/kg dexmedetomidine (Dexdomitor, 0.5 mg/mL, Zoetis Hellas) and 15 mg/kg ketamine (Imalgene 1000, 100 mg/mL, Merial, France) and instillation of 1–2 drops of topical anesthetic (proxymetacaine hydrochloride 0.5 %, Alcaine, Alcon Laboratories Hellas, Greece). The ocular surface and conjunctival fornix were cleaned and disinfected with a mild antiseptic solution containing 0.5% aqueous povidone–iodine, followed by placement of an eyelid retractor. Corneal diameter was measured to exclude animals with values differing from the mean**.**

**Figure 1:**
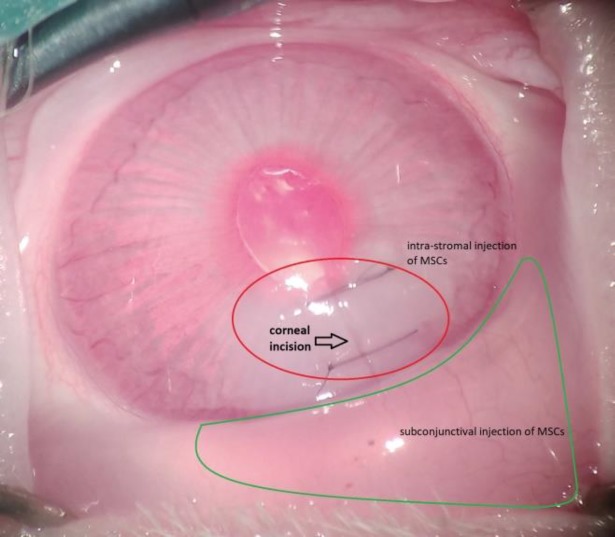
Image of a rabbit cornea immediately after adipose-derived mesenchymal stem/stromal cell (ADMSC) application. Corneal edema and conjunctival chemosis causing deformation of eye tissue and suture stretching are seen

A linear, full-thickness, 4-mm corneal incision was made with a disposable 15° ophthalmic knife adjacent to the superior corneolimbal junction and cautiously advanced centrally to avoid iris prolapse. The wound was securely sutured with two interrupted 10-0 nylon sutures (DemeTECH Co, Miami, The USA) parallel to the limbus and embedded (Figure 1). Wound integrity was tested using an ophthalmic strip containing fluorescein (Seidel test negative). The same investigator performed all surgeries to enhance reproducibility. Following suture setting and eye drop administration, animals were randomly selected to receive either ADMSCs (group 1; *n *= 16) or PBS (group 2, control; *n *= 16) *via* the same routes. In total, 2 × 10^6^ ADMSCs in 1 mL of PBS was applied to each cornea *via* three routes to increase the amount of ADMSCs infused and hence boost their effectiveness. A corneal intrastromal micropocket was created into the wound edges with an ophthalmic knife and 1/3 of the ADMSCs were infused. An ophthalmic microsurgical angular cannula was inserted into the cornea at the midstromal level of both sides of the incision, forming a deposit of ADMSCs. Furthermore, 1/3 of the ADMSCs were injected subconjunctivally. Finally, the remaining ADMSCs were applied locally on the wounded area (Figure 1). All the animals were administered a topical antibiotic (0.3% Ofloxacin; Oxatrex, Zwitter, Athens, Greece) and 1 % cyclopentolate HCL (Cyclogyl, Alcon, Greece) every 6 h on the first postoperative day, and then twice daily for the first week. Meloxicam (0.2 mg/kg subcutaneously; Metacam, 5 mg/mL, Boehringer Ingelheim, Germany) was also administered postoperatively and then once a day for the next 5 days. All sutures remained in place until photographs were taken. The length of corneal NV was measured from the limbal vascular plexus to its distant point (height). The contour of new sprouting vessels was also measured. All photographs were processed with Klonk Image Measurement version 16.1.14 (Image Measurement Co, Cheyenne, The USA). The animals were euthanized on day 14 when NV was prominently developed [29] and the eyeballs were enucleated.

Histology

The eyeballs were dissected and after formalin fixation dehydrated through 70%, 80%, and 95% alcohol followed by three changes of 100% alcohol and cleaning through two changes of xylene. They were dissected into 4-µm–thick slices, stained with hematoxylin and eosin (H&E) and subsequently histomorphometrically assessed by a pathologist.

**Immunohistochemistry**

The paraffin blocks with the eye tissue sections (4 μm) embedded, were used to implement immunohistochemistry with mouse anti-VEGF and anti-TNF antibodies (both from Abcam Biotechnology, Cambridge, The United Kingdom). Briefly, the eye tissue sections were incubated in a 3% H_2_O_2_ solution in methanol. Antigen recapture was performed by using 10 mM citrate buffer followed by incubation at 100°C. After incubation in blocking buffer, diluted primary antibodies were added to the sections for 1 h, followed by incubation with biotinylated secondary antibodies for 30 min. The expression of VEGF and TNF was revealed using the Peroxidase/DAB+ Dako Real Envision Detection System.

**Statistical analysis**

Using SPSS software version 20.0 for Windows (SPSS Inc., Chicago, IL, The USA) parametric statistical analyses were performed, after assessment of data normality with the Kolmogorov–Smirnov test. Independent-samples *t*-test was used to evaluate the differences in corneal NV distal point (height) and area. Levene’s test was performed to test the equality of group variances. Differences were considered significant when *p*-values were less than 0.05. 

## RESULTS

**Characterization of the administered ADMSCs**

**Figure 2:**
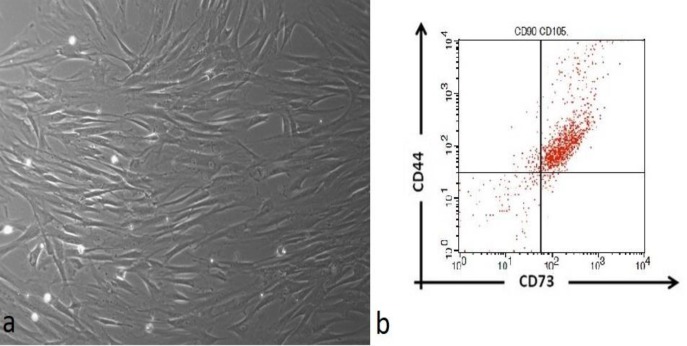
Morphology (a) and Immunophenotypic Characterization (b) of the Administered Adipose-Derived Mesenchymal Stem/Stromal Cells (ADMSCs)

**Figure 3:**
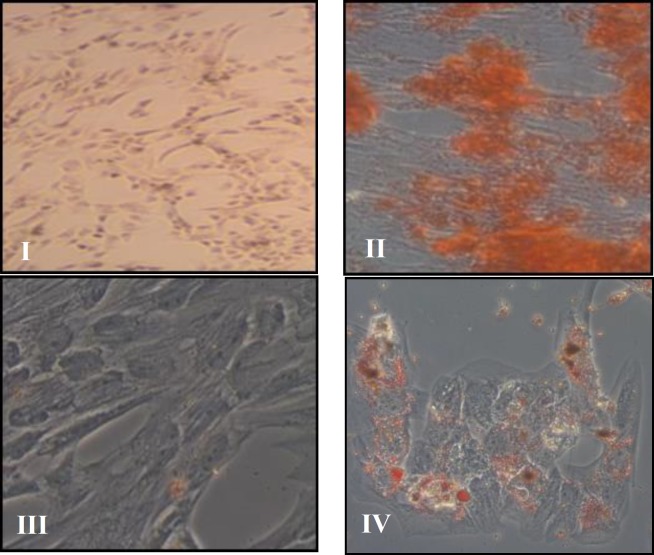
Morphological Depiction of Adipose-Derived Mesenchymal Stem/Stromal Cell (ADMSC) Differentiation. (I, Osteogenesis Negative Control; II, ADMSC Differentiation to Osteocytes; III, Adipogenesis Negative Control; IV, ADMSCs Differentiated to Adipocytes).

The cultured ADMSCs displayed a spindle-like, adult ADMSC morphology (Figure 2). Cells were additionally characterized based on CD44/CD73 markers detection (Figure 2). The ability of ADMSCs to differentiate into adipocytes and osteocytes was confirmed after completion of differentiation (Figure 3).

**The Effect of ADMSCs on Corneal Neovascularization**

**Figure 4:**
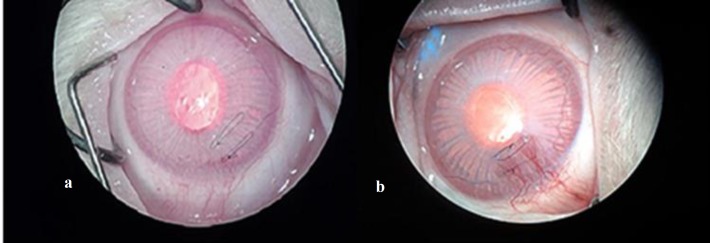
Representative images of angiogenesis in group 1 (adipose-derived mesenchymal stem/stromal cell [ADMSC]-treated) vs group 2 (control). (a) Eyes treated with ADMSCs exhibited limited (1.87 mm2 ± 0.90 mm2) neovascularization (NV) 14 days after ADMSC treatment. (b) Corneal NV in the control group 14 days after corneal injury (4.66 mm2 ± 1.74 mm2; p < 0.001, t-test). Corneal NV consisted of dense vessels emerging from pericorneal plexus capillaries, progressing and spreading over the distant suture, and covering a large region of the corneal surface

Neovascularization was observed in all injured corneas. Nevertheless, significantly less of the corneal surface was covered with neovascular tissue in ADMSC-infused eyes compared to control eyes (Table 1), as evidenced in photographs taken 14 days following suture placement. In the control, PBS-infused group, consequential compound, centripetal vessels emerged from the limbal area. In the ADMSC-treated group, the bulbar conjunctiva adjacent to the injury was mildly hyperemic, whereas a fully hyperemic pattern was established in the control group (Figure 4). 

**Table 1:** Corneal Neovascularization in Eyes Treated With Adipose-Derived Mesenchymal Stem/Stromal Cells and Those of the Control Group

Group	Measurement	Mean ± SD	Standard error	95 % CI of the mean	p-value
ADMSC-treated	Distal point (mm) [30]	0.98 ± 0.30	0.07	0.83–1.12	p < 0.001
	Area (mm^2^)	1.87 ± 0.90	0.21	1.44–2.30	
Control	Distal point (mm) [30]	2.88 ± 0.58	0.15	2.58–3.18	
	Area (mm^2^)	4.66 ± 1.74	0.44	3.78–5.54	

Abbreviations: ADMSC, adipose-derived mesenchymal stem/stromal cell; SD: standard deviation; CI: confidence interval (p < 0.001, t-test).

**Figure 5:**
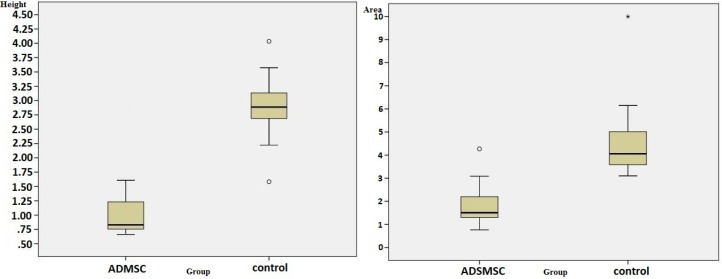
Extent of corneal neovascularization (NV) in group 1 (adipose-derived mesenchymal stem/stromal cell [ADMSC]-treated) vs. group 2 (control) 14 days after corneal injury. Interquartile range demonstrating the corneal NV distal point (height) and area in the ADMSC-treated group vs. the control group. Differences were considered significant at p < 0.001, t-test)

No corneal opacities or scars were observed in either group as the wound was surgically incised and sutured without delay. No immune rejection occurred in any of the eyes of the ADMSC-treated group. The area and distal point of corneal NV in both groups are depicted in Figure 5. The corneal NV distal point from limbus (height) in group 1 (ADMSC-treated) (0.98 ± 0.3 mm) was significantly lower than that in group 2 (control) (2.88 ± 0.58 mm, *p *< 0.001). Furthermore, the corneal NV surface was significantly reduced in group 1 (1.87 ± 0.9 mm^2^) compared to that in group 2 (control) (4.66 ± 1.74 mm^2^, *p* < 0.001) (Table 1).

**Histopathological and Immunohistochemical Findings**

**Figure 6:**
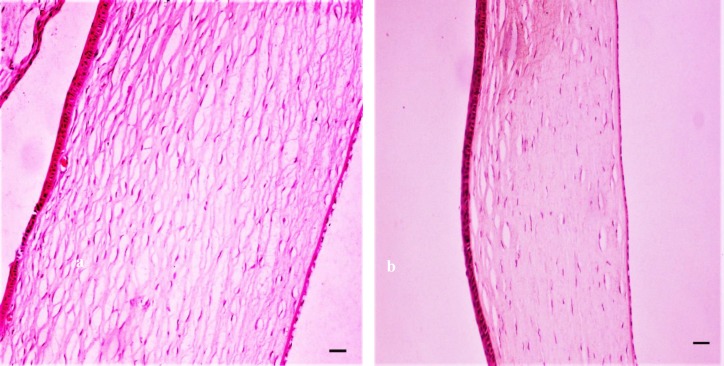
Moderate edema was observed in the eyes of the control group (a). H&E staining showing that the corneas of adipose-derived mesenchymal stem/stromal cell (ADMSC)-treated eyes were normal 14 days after injury, H&Ε, x100 (b). Bar = 100 μm

In the control group, the injured corneas exhibited severe NV and disorganized stroma with severe edema. Although mild edema was noted in the ADMSC-treated group following administration, the surface appeared smooth and exhibited intact, almost physiologically normal tissue (Figure 6).

**Figure 7:**
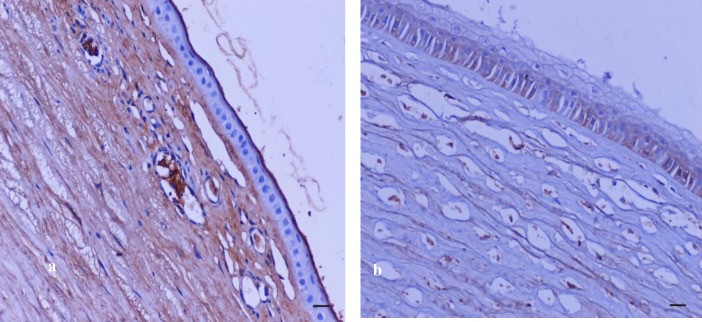
Vascular endothelial growth factor (VEGF) immunostaining of rabbit corneal sections. VEGF staining was intense in control eyes presenting corneal NV, x200 (a), whereas no capillaries were detected in adipose-derived mesenchymal stem/stromal cell (ADMSC)-treated corneas, x200 (b); bar = 50 μm

**Figure 8:**
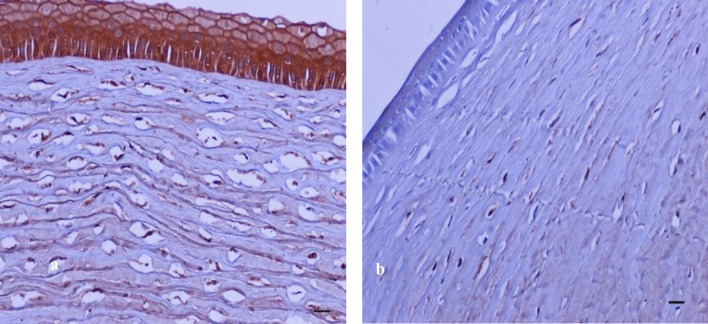
Tumor necrosis factor (TNF) immunolocalization in epithelial and stromal cells in eyes from the control group, x200 (a). No TNF-positive cells were present in adipose-derived mesenchymal stem/stromal cell (ADMSC)-treated eyes, x200 (b). Bar = 50 μm

Immunostaining for VEGF and TNF was performed to identify the presence of corneal NV and inflammation, respectively. In contrast to that observed for control eyes, no VEGF staining was detected after induction of corneal injury with ADMSC treatment, indicating the beneficial effect exerted by administered cells in decreasing pathological NV (Figure 7). Additionally, TNF staining was also not detected in ADMSC-treated corneas, whereas its expression was detected in corneal epithelium and stroma of eyes in the control group (Figure 8). No TNF-positive cells were represented, demonstrating a limited inflammatory environment, thereby confirming the results of the H&E staining.

## DISCUSSION

In the present study, we used an injury-induced NV rabbit model to examine the anti-angiogenetic effects of ADMSCs in the cornea. We evaluated their ability to reduce corneal NV when administered via three combined routes. Our results indicate that the area of corneal NV in the ADMSCs-treated eyes is significantly reduced compared to the control group following injury to cornea. 

In 2011, approximately 150,000 penetrating keratoplasty surgeries related to ocular surface pathology were performed worldwide [31]. Neovascularization may compromise the cornea’s normal immunological privilege, leading to rejection and subsequent graft failure [32]. Corneal surgery and other injuries may induce the emergence of new vessels from the limbal arcade, involving the whole cornea, which may subsequently compromise vision. 

Mesenchymal stem/stromal cells are multipotent cells with antiangiogenic properties [23]. Oh et. al demonstrated that infusion of human MSCs into mice greatly affected survival of allogeneic corneal transplants [33]. The results of the present study indicated that allogeneic MSCs significantly reduce corneal NV when applied in a rabbit model of corneal injury. 

Different routes of administration have been used for MSC transplantation, such as intrastromal injection [34], subconjunctival administration [35], systemically [36], with nanofiber scaffolds [37], and into the anterior chamber [28]. Ghazaryan et al. suggested that subconjunctival injection overcomes the hurdles of amniotic membrane-associated MSC transplantation and reduces VEGF levels, resulting in better outcomes [38]. In our model, MSC applied by three different routes effectively inhibited the formation of new vessels when administered immediately after incision. 

Mesenchymal stem/stromal cells secrete soluble molecules that inhibit angiogenesis and reduce the levels of VEGF [23] via a paracrine signaling process, with a concomitant upregulation of TNF-stimulated gene-6 (TSG-6) [39]. Several studies investigating vascular and arterial diseases have shown that MSC paracrine activity may be mediated through exosome secretion [30, 40, 41]; however, the associated mechanisms remain unknown.

Application of MSCs using local subconjunctival injection stimulates the therapeutic potential of wounded corneas without corneal inflammatory cell infiltration [35]. Song et. al. found that intravenous injection (systemic administration) of bone marrow-derived MSCs induced a prominent reduction in corneal NV and levels of VEGF [42]. In this study, we administered ADMSCs by infusion (local administration). Consistent with the findings of Song et al, the extent of NV was significantly lower in ADMSC-treated corneas than controls, indicative of reduced VEGF levels. Immunohistochemical data showed that VEGF and TNF were expressed in the corneal epithelium and stroma of the control group, but not in those of the ADMSC-treated group. In agreement with our findings, other studies have also reported that TNF is associated with corneal NV through induction of vasodilatation and leukocyte extravasation [43-45].

Inoculation of MSCs after chemically induced corneal injury has also been reported to be more effective than topical application, possibly because direct contacts amongst cells enhance the anti-inflammatory properties of cytokines following MSC inoculation [23]. Transplanted MSCs may differentiate into keratocytes or other cell types such as epithelial progenitor cells, corneal endothelial cells, or myofibroblasts. An alternative mechanism is that viable epithelial cells around the wound would be induced to multiply and contribute to healing, masking the deficits of the limbus. The latter effect of MSCs can reportedly be mediated by growth factors such as VEGF, epidermal growth factor (EGF) and TGF-β [19, 23, 34]. Based on the above data, we suggest that MSCs can be used as an alternative therapy in cases where other antiangiogenic therapies have failed.

Our study indicated that rabbit ADMSCs preserve their antiangiogenic properties for up to 5 duplications. Wagner et al [46]. reported that MSC self-renewal rate declines after three months in cell culture, eventually reaching senescence (Hayflick limit). This is a key factor in the use of MSCs, as they should be at an early stage to exert their unique therapeutic effects. A recent study [28] showed that application of young MSCs (after three passages) was effective to inhibit corneal scarring after penetrating injury in rat corneas. ADMSCs also preserve the potential to differentiate through a well described procedure into corneal epithelial cells, demonstrating their ability for biomedical engineering [47, 48].

Numerous studies on animal models of chemically induced injury have demonstrated the beneficial effect exerted by MSCs in corneal wound healing [49, 50]. However, little evidence exists regarding mechanically-induced corneal injuries which form a sizeable part of *in vivo* corneal pathology [28].

In our experimental setting, we used a well-described model of penetrating injury to induce corneal NV. We investigated alternative approaches by combining three types of local MSC application. Our study supports that ADMSCs infusion via three alternative routes may have a beneficial role in corneal NV through modulation of VEGF and TNF secretion. Additionally, ADMSCs can be readily obtained and transplanted, at least in our experimental model (rabbit). To the best of our knowledge, this is the first time that ADMSCs have been applied using three different paths and techniques to successfully reduce and control corneal NV following mechanical corneal injury.

A limitation of our study was restricted number of rabbits. The corneal incision was promptly sutured and no scar tissue was produced, failing to demonstrate ADMSCs efficacy in corneal transparency following wound healing. Another limitation was failure to identify whether the transplanted cells were differentiated into corneal host cells.

Further studies with sufficient number of animals are required for the assessment of MSCs efficacy in ocular pathology. Additional clinical trials must be meticulously performed to assess the minimum effective number of MSCs and possible adverse events before the patient’s own MSCs may be transferred in selected cases when the patient’s vision is compromised [51].

## CONCLUSION

Local ADMSC administration can lead to significant inhibition of corneal NV in traumatically induced corneal NV in rabbits. To elucidate the practical clinical utility, further research is needed to standardize the effective ADMSC dosage, frequency of administration and ideal mode of application.

## DISCLOSURE

Ethical issues have been completely observed by the authors. All named authors meet the International Committee of Medical Journal Editors (ICMJE) criteria for authorship of this manuscript, take responsibility for the integrity of the work as a whole, and have given final approval for the version to be published. No conflict of interest has been presented. Funding/Support: None. The datasets analyzed during this study are available from the corresponding author on reasonable request.
